# Disease association and comparative genomics of compositional bias in human proteins

**DOI:** 10.12688/f1000research.129929.1

**Published:** 2023-02-20

**Authors:** Christos E. Kouros, Vasiliki Makri, Christos A. Ouzounis, Anastasia Chasapi

**Affiliations:** 1 BCCB-AIIA, School of Informatics, Aristotle University of Thessaloniki, Thessaloniki, Greece; 2 BCPL, Chemical Process & Energy Resources Institute, Centre for Research & Technology Hellas (CERTH), Thessaloniki, Greece

**Keywords:** disease-associated gene, low complexity, compositional bias, intrinsically disordered protein (IDP), intrinsically disordered region (IDR), phylogenetic profile, human genome, human disease

## Abstract

**Background:** The evolutionary rate of disordered proteins varies greatly due to the lack of structural constraints. So far, few studies have investigated the presence/absence patterns of intrinsically disordered regions (IDRs) across phylogenies in conjunction with human disease. In this study, we report a genome-wide analysis of compositional bias association with disease in human proteins and their taxonomic distribution.

**Methods: **The human genome protein set provided by the Ensembl database was annotated and analysed with respect to both disease associations and the detection of compositional bias. The Uniprot Reference Proteome dataset, containing 11297 proteomes was used as target dataset for the comparative genomics of a well-defined subset of the Human Genome, including 100 characteristic, compositionally biased proteins, some linked to disease.

**Results: **Cross-evaluation of compositional bias and disease-association in the human genome reveals a significant bias towards low complexity regions in disease-associated genes, with charged, hydrophilic amino acids appearing as over-represented. The phylogenetic profiling of 17 disease-associated, low complexity proteins across 11297 proteomes captures characteristic taxonomic distribution patterns.

**Conclusions:** This is the first time that a combined genome-wide analysis of low complexity, disease-association and taxonomic distribution of human proteins is reported, covering structural, functional, and evolutionary properties. The reported framework can form the basis for large-scale, follow-up projects, encompassing the entire human genome and all known gene-disease associations.

## Introduction

### Disordered proteins exhibit specific patterns at the sequence level

The classical view that protein function requires a defined three-dimensional (3D) structure has been challenged by recent developments where many proteins and protein regions are shown to perform distinct biological functions, despite their propensity for disordered conformations. These intrinsically disordered proteins (IDPs) and intrinsically disordered protein regions (IDRs) are defined as lacking a precise 3D folding pattern. The difference between ordered and disordered proteins is already reflected at the primary structure level, with IDPs being characterized by regions typically enriched in specific amino acids, resulting in an overall low sequence complexity. Specifically, IDPs and IDRs contain substantially fewer residues that promote order (typically C, F, I, L, N, V, W and Y) and are substantially enriched in residues that promote disorder (typically A, E, G, K, P, Q, R, and S) ( Williams *et al.*, 2000; Dunker *et al.*, 2001; Harbi *et al.*, 2011).

### IDP/IDR identification

With the increasing number of predicted and experimentally validated IDPs and proteins containing IDRs, disordered proteins and regions are no longer considered as exceptions, but rather the object of extensive study with regard to their structure and function. A wide range of disorder predictors has been successfully developed over the past years, adopting different approaches such as Compositional Bias Detection ( Harrison, 2021; Promponas *et al.*, 2000; Wootton & Federhen, 1993), residual energy-based disorder prediction ( Dosztanyi *et al.*, 2009, 2005) and others ( Linding *et al.*, 2003; Tang *et al.*, 2021; Walsh *et al.*, 2012; Wang *et al.*, 2016; Zhang *et al.*, 2012). Integrative tools have made their appearance, such as MobiDB-lite ( Necci *et al.*, 2017), a data fusion tool making use of eight distinct predictors. The prediction accuracy of such tools varies greatly, with deep learning-based methods typically outperforming methods based on physicochemical characteristics ( CAID Predictors *et al.*, 2021). DisProt, a manually curated, dedicated database for IDPs ( Sickmeier *et al.*, 2007) has developed into the main resource for IDP/IDR information ( Hatos *et al.*, 2019; Quaglia *et al.*, 2022).

### IDPs, phylogeny and disease

Multiple computational and experimental analyses of a wide range of species at the genome level have established widespread presence of intrinsic disorder across the tree of life ( Hatos *et al.*, 2019; Ntountoumi *et al.*, 2019; Peng *et al.*, 2015; Ward *et al.*, 2004). In fact, proteins at all taxonomic levels, including viruses, exhibit noticeable intrinsic disorder that apparently increases with organism complexity. Disorder presence is particularly prominent in eukaryotes, in which at least half of their genome-encoded proteins possess long IDRs ( Ahrens *et al.*, 2017; Basile *et al.*, 2019; Peng *et al.*, 2015; Ward *et al.*, 2004; Xue *et al.*, 2012). This high prevalence of IDPs and IDRs in eukaryotes indicates that key functions, such as cell signalling and regulation, are transiently associated with intrinsic disorder in nucleated cells ( Bürgi *et al.*, 2016; Tantos *et al.*, 2012).

The same trend holds for an ever-increasing emergence of disease-associated genes in more recent speciation events ( Dickerson & Robertson, 2012; Lopez-Bigas & Ouzounis, 2004), raising the question whether specific residues can be directly implicated in particular diseases. A correlation between intrinsic disorder and various human diseases such as cancer, diabetes, amyloidosis, and neurodegenerative diseases has already been established in specific cases ( Choudhary *et al.*, 2022; Monti *et al.*, 2021, 2022), and is emerging as a significant biomedical research endeavour.

Due to a lack of structural constraints, the evolutionary rate of disordered proteins varies, with some IDPs/IDRs being highly conserved while others appearing particularly diversified ( Brown *et al.*, 2011; Khan *et al.*, 2015; Xue *et al.*, 2013). So far, few studies have investigated the phylogenetic profiling of IDRs in conjunction with human disease ( Pajkos *et al.*, 2020). To assess this hypothesis, we use a curated list of 100 annotated proteins from the human genome with well-characterised compositionally biased regions (CBRs) ( Mier *et al.*, 2020), as a first step for the comparative genomics of compositionally biased genes, some of which are in fact disease associated. We identify those instances known to be linked with human disease and assess their phylogenetic depth. This framework, with human queries against multiple species, forms the basis for follow-up, large-scale studies that would encompass the entire human genome and all known gene-disease associations.

## Methods

### Data compilation

The Human Genome protein set recorded in the Ensembl database (GRCh38.p13) was retrieved, containing ~119K gene transcripts to be used as reference ( Yates *et al.*, 2016).

For the disease mapping on gene transcripts, the DISEASES database was chosen, which integrates disease-gene associations derived from text mining, as well as manually curated disease–gene associations, cancer mutation data, and genome-wide association studies from existing databases ( Pletscher-Frankild *et al.*, 2015). Specifically, the “Knowledge channel” was selected, containing manually curated associations from GHR ( Koos & Bassett, 2018) and UniProtKB ( The UniProt Consortium *et al.*, 2022), a total of 7269 disease-gene, high-confidence associations.

Disease associations are provided with the use of Disease Ontology identifiers (DOID) ( Schriml *et al.*, 2019). For each entry of the Ensembl dataset, DOIDs were mapped from the DISEASES knowledge channel dataset and added to the header description of the corresponding gene transcript.

For phylogenetic analysis, the Uniprot Reference Proteome (URP) dataset was selected, containing a total of 11297 proteomes, excluding viruses. The URP set has been selected manually and algorithmically among all proteomes, to provide broad coverage of the tree of life, representing the taxonomic diversity found within UniProtKB and including the proteomes of well-studied model organisms and other proteomes of interest for biomedical research ( Chen *et al.*, 2011). Specifically, the URP (version: Reference_Proteomes_2022_04) contains 349 Archaeal, 8763 Bacterial and 2185 Eukaryotic proteomes.

The low complexity query set investigated both for its disease association and phylogenetic depth was previously recorded ( Mier *et al.*, 2020) and contains 100 human proteins with characteristic compositional bias.

### Data transformation

The computational pipeline **cogent_utils,** part of CGG toolkit v1.0.1. (Vasileiou et al, submitted), was used to create a CoGenT-style sequence collection ( Janssen *et al.*, 2003) from Ensembl GRCh38.p13 as well as the URP, selected as a robust and convenient identifier encoding scheme both for human interpretation and programming convenience. Specifically, cogent_utils enables header modification for all entries of FASTA sequence files, based on user-defined criteria. Below we present the example of the oleosine protein of *Camellia sinensis*, as it appears originally in URP and after cogent_utils transformation:

*URP original header*

>tr|A0A7J7IAQ7|A0A7J7IAQ7_CAMSI Oleosin OS=Camellia sinensis OX=4442 GN=HYC85_002860 PE=3 SV=1

*Modified header*

>UP000593564-00004442-Came_sine-22-000001-E-000699 tr|A0A7J7IAQ7|A0A7J7IAQ7_CAMSI Oleosin OS=Camellia sinensis OX=4442 GN=HYC85_002860 PE=3 SV=1

The first part of the header has been added, and corresponds to the following format: [URP identifier]-[NCBI Taxonomy ID]-[organism name]-[URP year release]-[proteome counter]-[taxonomic domain]-[protein counter].

**MagicMatch** v1.0.1 ( Smith *et al.*, 2005) was used for sequence matching across databases to verify the identity of the reference proteome collection against the modified identifier space.

### Masking, searching, phylogenetic profiling

For the detection of compositional bias as a proxy for low-complexity sequence tracts, we deployed **CAST v1.0.1** ( Promponas *et al.*, 2000), for all protein sequences of the human genome. The CAST algorithm was applied on the DOID annotated Ensembl FASTA format dataset using default parameters, i.e. threshold score 40 for reported regions. The outcome of the analysis were 2 files dividing the original dataset; one containing all entries where low complexity regions were detected and one containing all remaining entries.

Searching with query datasets against Proteomes for the creation of phylogenetic profile patterns was performed with **DIAMOND** blastp ( Buchfink *et al.*, 2021), using the URP dataset as target database and adjusting the alignment algorithm to enable compositional bias statistics (option: --comp-based-stats 3), conditioned on sequence properties ( Yu & Altschul, 2005). All hits considered as significant recorded an E-value<0.001 and exhibit sequence similarities of 21% and above.

**Figure 1.**
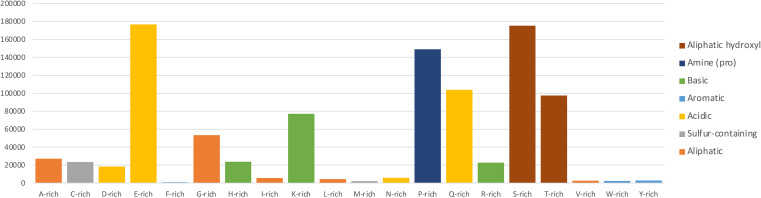
Compositionally biased protein regions identified using CAST on the Ensembl human genome. Sum of CAST scores for all compositionally biased occurrences by amino acid, normalised with respect to general amino acid frequency in the human genome (i.e. total score/frequency). The column colour corresponds to an amino acid classification according to the chemical nature of their side chains ( Katchalski-Katzir *et al.*, 2006).

For the calculation of amino acid frequencies across the Ensembl protein set, the BioPython Bio.SeqUtils.ProtParam module ( Cock *et al.*, 2009) was used, which takes input files of sequences (typically FASTA or FASTQ), counts all the letters in each sequence, and returns a summary table of their counts and percentages. The output was used for data normalisation as explained in Figure 1.

The phylogenetic profile heatmap ( Figure 4) was produced using the heatmap3 R library ( Zhao *et al.*, 2014) with default dissimilarity matrix calculation parameters.

The 2×2 chi-square test, comparing low complexity presence in protein transcripts and disease-association ( Table 1) was performed with 0.01 significance threshold and no Yates continuity correction.

**Table 1.** Contingency table between disease association in genes presenting compositionally biased regions versus genes without any detectable compositional bias.

	Low complexity	High complexity	
**Non-disease**	36250	62827	99077
**Disease**	1845	1780	3625
	38095	64607	102702

**Lifemap**, an interactive cartography-type tool to explore the NCBI taxonomy was chosen for the visualisation of the taxonomic distribution of data subsets ( de Vienne, 2016). For each visualisation, a list of the NCBI IDs of interest were used as input for the tool, which were retrieved, in each case, from the phylogenetic profiling hit list. NCBI taxonomy ID visualisations are provided for all UPR hits of ALG13, SIX3 and RP9 ( Figure 5).

## Results

### Disease association across the human genome

The dataset upon which all transformations and analyses were performed was the Ensembl Human Genome export (GRCh38.p13), containing 119068 gene transcripts. The dataset was annotated with regard to disease association, using curated associations from GHR and UniProtKB, which are indexed in the DISEASES database ( Pletscher-Frankild *et al.*, 2015). Of these, 3625 transcripts are confidently associated with disease, whereas the remaining 115443 are not verified for any strong disease association in the “knowledge channel” of DISEASES.

To remove noise, e.g. putative or alternative mini-transcripts (some with multiple stop codons), the Ensembl dataset was filtered and all transcripts with length <80 amino acid residues were removed, with the exception of short transcripts with at least one disease (i.e. DOID) association. The filtered set contains 102702 transcripts, which include all 3625 instances associated with disease ( Table 1).

### Compositional bias and human disease

For the evaluation of low complexity presence in the transcripts of the human genome we performed compositional bias detection using CAST ( Promponas *et al.*, 2000). Out of the 102702 transcripts of the filtered Ensembl human genome dataset, compositional bias was detected in 38095 instances, with at least one compositionally biased sequence tract. Cross-evaluation of compositional bias and disease-association presence in the dataset using chi-square test of independence, revealed a significant bias towards low complexity regions in disease-associated, X ^2^ (1, N = 102702) = 306.8467, p-value < 0.00001 ( Table 1). This significant pattern alone provides a strong indication for the involvement of low complexity in human disease on genome scale, seen here for the first time, complementing previous, well-established classifications of protein structure and function ( Ouzounis *et al.*, 2003).

Examination of the low complexity gene dataset features highlighted the significant divergence among amino acid-related, low complexity frequencies. Figure 1 shows the amino acid-specific rich regions, expressed by the sum of CAST scores for each compositionally biased region and normalised with respect to general amino acid frequency in the human genome as calculated from the filtered Ensembl dataset using Biopython protein analysis modules ( Cock *et al.*, 2009). Charged, hydrophilic residues appear over-represented, while hydrophobic, order-promoting amino acids are less frequent, in agreement with what is known about IDP/IDR composition ( Williams *et al.*, 2000; Dunker *et al.*, 2001; Harbi *et al.*, 2011). The striking over-representation of serine/threonine (S/T) tracts, along with glutamate/glutamine (E/Q) and proline (P) followed by lysine (K) is indicative of the main residue types that might affect functional properties of human proteins, including their potential association with known phenotypes, such as polyglutamine tracts with neurodegenerative diseases ( Bunting *et al.*, 2022).

**Figure 2.**
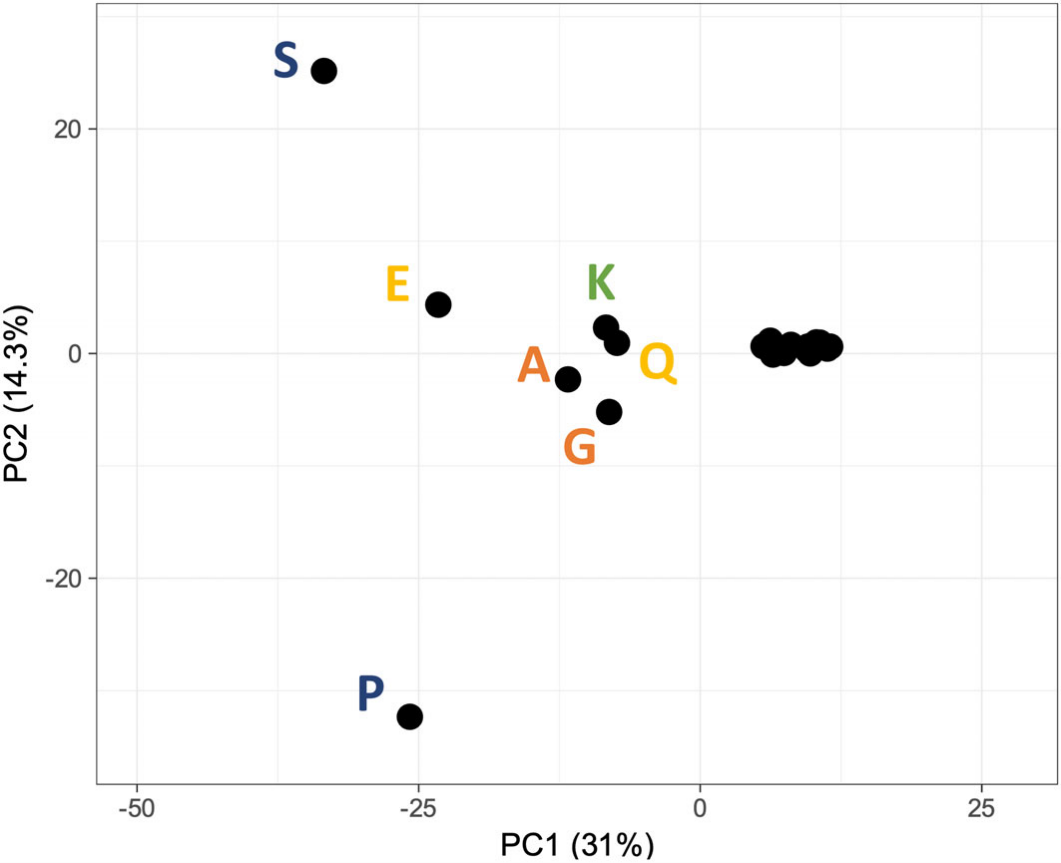
PCA analysis of DOID correlation to proteins with low complexity regions, across the human genome. The colour coding of each amino acid is the same as Figure 1 and reflects the chemical nature of their side chains. DOID=Disease Ontology identifier.

For the assessment of the relationship among disease association and compositional bias across the human proteome, the associated DOID vector for each amino acid enriched region was used as a multidimensional clustering parameter for Principal Component Analysis (PCA) ( Figure 2). Consistent with the above, the presence of amino acid types in low complexity regions (e.g. S, E, P, Q) exhibit the highest contribution to the main principal components with regard to disease association, thus amplifying the link between low complexity and disease and establishing a direction for further study.

### Phylogenetic profiling of disease-associated LC proteins

**Figure 3.**
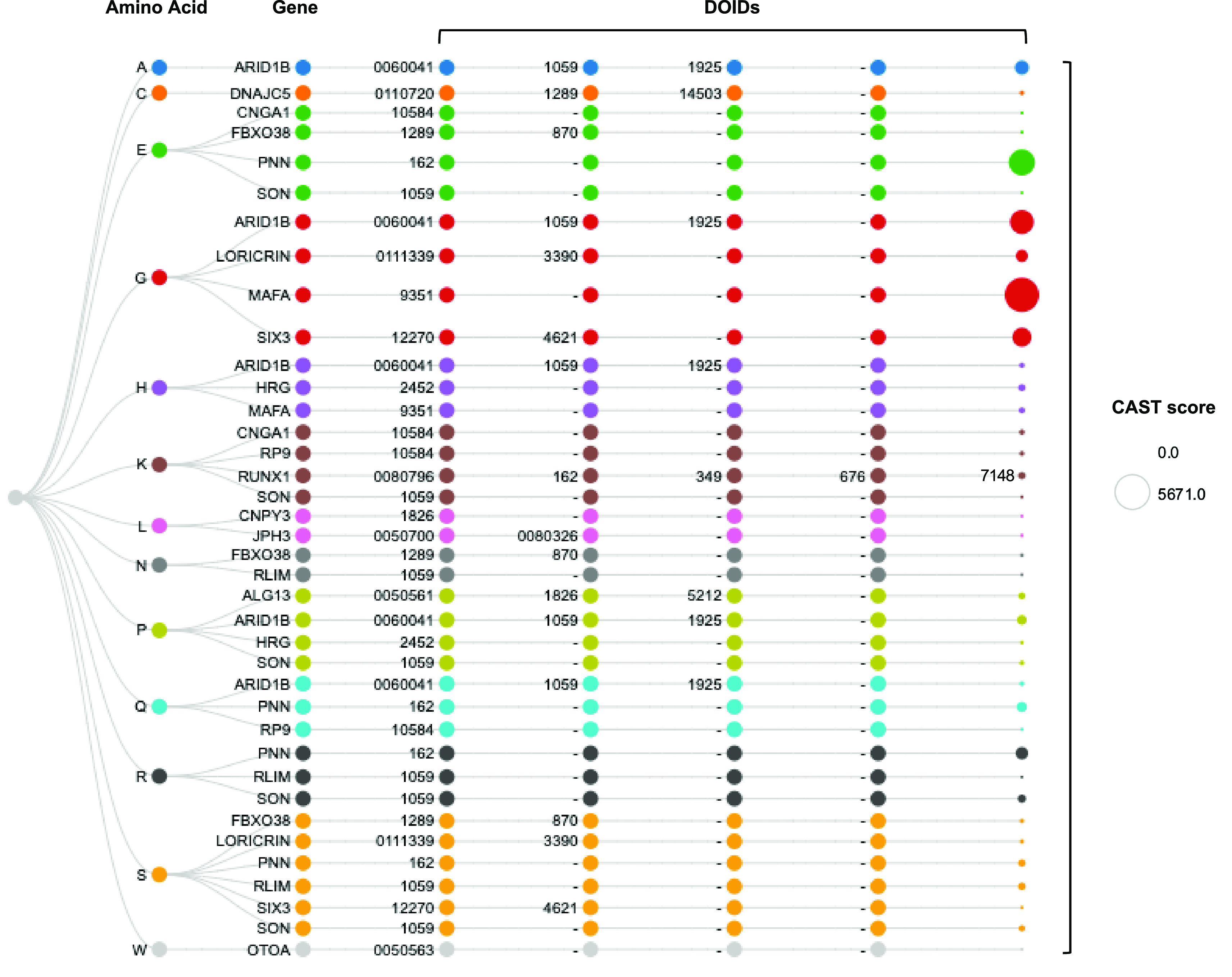
Compositionally biased regions of the 100-gene subset associated with disease, linked to associated DOIDs. The first column corresponds to the amino acid that appears to have enriched presence in each case. The last column’s size is proportional to the sum of CAST scores for amino acid rich regions in each gene. Although specific genes have originally been listed as exemplary for one compositional bias type, in this analysis they can be observed more than once along their DOID associations, as the result of CAST analysis for the derivation of total scores. DOID=Disease Ontology identifier.

To further our investigation into the phylogenetic depth of low complexity proteins with or without known disease associations, we selected a published list of 100 human proteins with well-characterised compositionally biased regions ( Mier *et al.*, 2020). The proteins were mapped to the enriched human genome datasets derived from Ensembl. Out of the 100 proteins in this curated dataset, 17 are confidently associated with disease, with one or more associated DOIDs, covering a wide range of disorders from metabolic and cardiovascular diseases to autoimmune conditions and cancer ( Figure 3).

To examine in more detail the emergence of compositional bias for the curated dataset of 100 human proteins as an exemplary case, protein sequence alignment was performed against the URP dataset. Homologues were detected in >11000 species, with just 269 cases not containing any of these regions, largely corresponding to Archaeal and Bacterial taxa. This preliminary, targeted comparative analysis using a limited query of 100 human proteins is a first glimpse into the dynamics of compositional bias across phylogenies. Our ongoing effort to investigate the presence of compositional bias and the connection to human disease will assess these discovered phylogenetic patterns across the entire human genome in the near future. The complete phylogenetic profiling matrix is provided as *Extended data* ( Chasapi, 2022).

**Figure 4.**
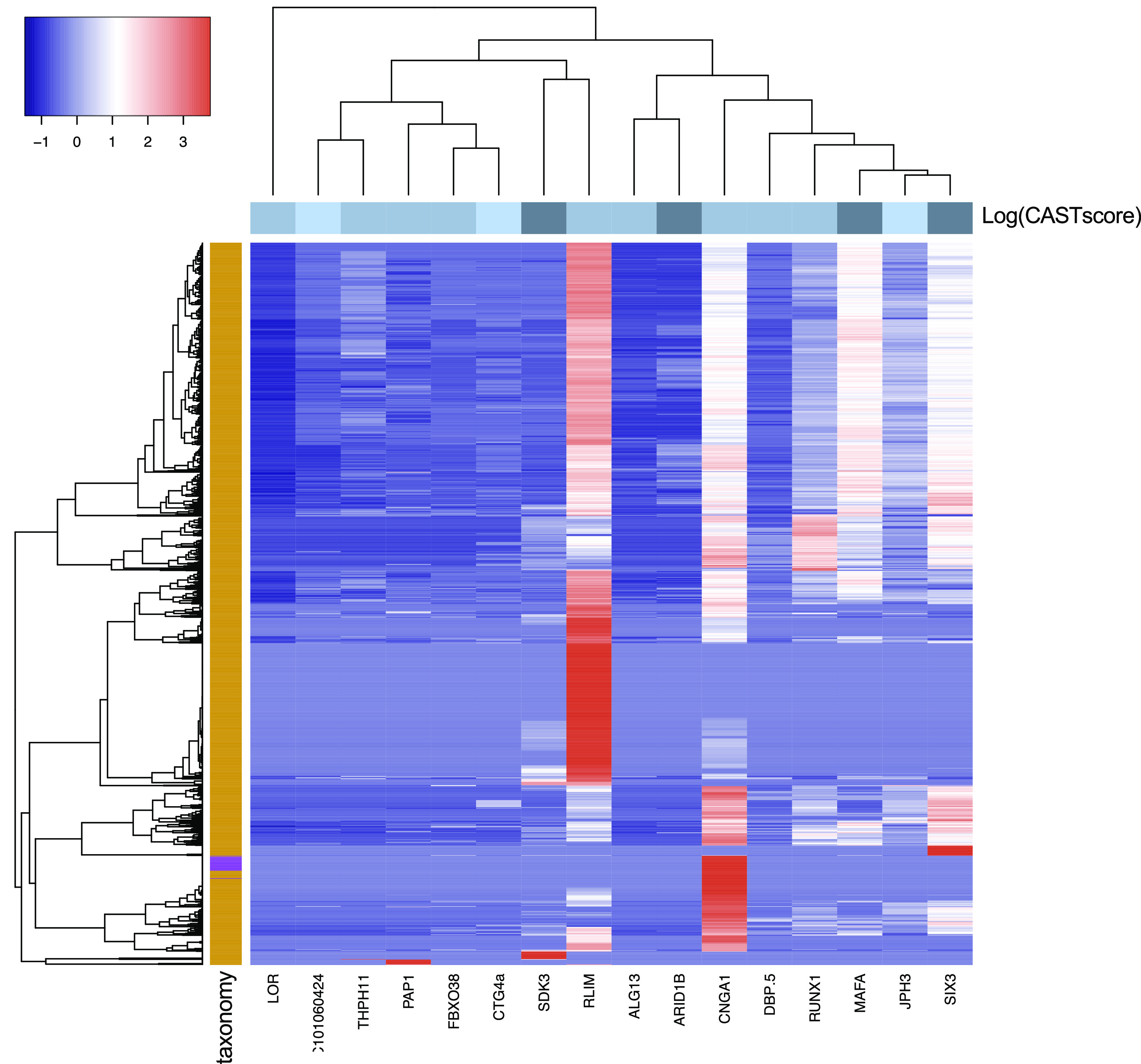
Phylogenetic profile of the 100-gene subset with confident disease associations. The heatmap range reflects the dissimilarity matrix of the plotted values. The row side colours correspond to the log of the sum of CAST scores for all detected compositionally biased regions for each protein (darker colour indicates higher sum of CAST scores). The column side colours indicate the taxonomic level of each target proteome (orange = eukaryotes, purple = bacteria). DNAJC5 is not displayed.

Focusing on the 100-gene subset with confident disease associations, most disease-associated genes had detectable homologues across Eukaryotic organisms, with only a few, scarce Bacterial hits ( Figure 4). An exception is the DnaJ heat shock protein family (Hsp40) member C5 (DNAJC5) which exhibits an extended phylogenetic depth, covering 86% of the URP (i.e. 9751 proteomes), verifying the observation as a well-known, abundant domain ( Stetler *et al.*, 2010; Qiu *et al.*, 2006).

The remaining, 16 disease-associated genes were detected in 1350 proteomes with one or several hits. Figure 4 shows the phylogenetic profile map of these genes across the URP target proteome set. Most genes display homologues in higher eukaryotic organisms, whereas, with the exception of the E3 ubiquitin-protein ligase RLIM, almost no homologous genes are detected in plant genomes. Similarly, the subunit of the rod cyclic GMP-gated cation channel (CNGA1) is the only query gene with ion channel homologues in ciliates and fungi, with the exception of the Ascomycota. In the case of genes with an overall high CAST score, there seem to be more sequence hits, both in number and in taxonomic distribution. This can be, in part, due to the sequence alignment analysis which was tailored to compositionally biased sequences, thus increasing hit sensitivity.

This comparative genomics framework is a useful tool both for the investigation of tendencies among gene sets confidently associated with diseases, containing compositionally biased regions, as well as for the identification of specific taxonomic signatures for each gene. A selected number of specific cases are reviewed below.

### ALG13 has a restricted phylogenetic depth

**Figure 5.**
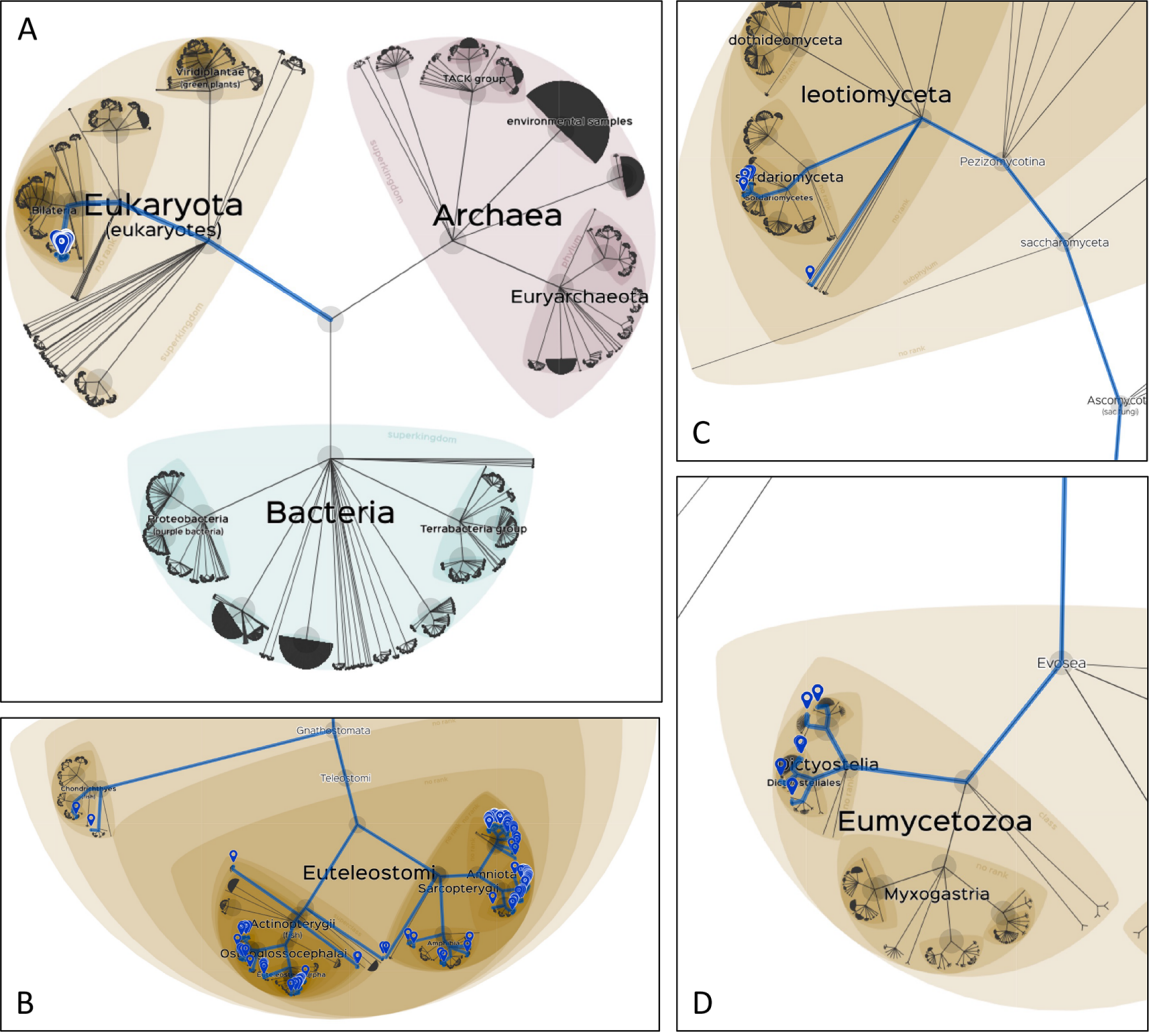
Taxonomic distribution of sequence alignment hits for selected genes, across the URP proteome dataset. A) a broad view of the tree of life, visualised be Lifemap ( de Vienne, 2016). ALG13 hits are highlighted in blue. B) A zoomed in view of all ALG13 hits. C) SIX3 hits that belong to the Ascomycota phylum and are not found for any other gene of the dataset. D) RP9 hits that correspond to the Dictyostelia clade and are not found for any other gene of the dataset.

The protein encoded by ALG13 is a subunit of a bipartite UDP-N-acetylglucosamine transferase, which heterodimerizes with asparagine-linked glycosylation 14 homolog to form a functional UDP-GlcNAc glycosyltransferase that catalyses the second sugar addition of the highly conserved oligosaccharide precursor in endoplasmic reticulum N-linked glycosylation. ALG13 has been associated with several disease conditions including developmental and epileptic encephalopathy as well as genetic intellectual disability ( Epi4K Consortium & Epilepsy Phenome/Genome Project, 2013; Bissar-Tadmouri *et al.*, 2014; Ng *et al.*, 2020). ALG13 homologs are detected in 248 proteomes. Moreover, all hits correspond to higher Eukaryotes, specifically to the infraphylum Gnathostomata, including mostly Euteleostomi representatives. Figure 5A shows a general view of the tree of life, highlighted for species where ALG13 homologue hits were retrieved, whereas Figure 5B provides a closer look of the same result. The restricted phylogenetic depth of ALG13 may indicate that the interaction pathways including ALG13 are restricted to functions specific to bony vertebrates, a hypothesis that can be assessed by jointly analysing all participating proteins for their evolutionary emergence.

### SIX3 has a unique conservation signature

SIX Homeobox 3 (SIX3) encodes a member of the sine oculis homeobox transcription factor family. The expressed protein plays a role in brain and eye development, and its mutations are associated with Holoprosencephaly and Schizencephaly abnormalities ( Wallis *et al.*, 1999, 3; Hehr *et al.*, 2010, 3). SIX3 homologues were detected in 869 reference proteomes, including filamentous ascomycetes proteome sequences in which SIX3 is the only disease-associated gene with significant hits ( Figure 5C). A follow-up study could further investigate this distinct conservation pattern.

### RP9 is uniquely matched in Dictyostelia

Retinitis Pigmentosa 9 (RP9 or PAP1) is thought to be a target protein for the PIM1 serine/threonine protein kinase. The protein localises in nuclear speckles and has a role in pre-mRNA splicing. Mutations in the RP9 gene result in autosomal dominant retinitis pigmentosa ( Maita *et al.*, 2004; Keen *et al.*, 2002). The comparative genomics analysis of RP9 presence detects homologues in 507 species, including all representatives of the Dictyostelia clade, that were uniquely matched to RP9 among all disease genes ( Figure 5D). *Dictyostelium discoideum*, the most studied representative of Dictyostelia (i.e. dictyostelid cellular slime molds), has been used extensively as model organism for cell communication, differentiation, and programmed cell death studies ( Kawabe *et al.*, 2019; Strassmann *et al.*, 2000). The specific presence of RP9 homologues in Dictyostelia including D. *discoideum*, raises questions about their specific roles in this taxon and the possibility that functional analysis can shed further light into the human disease.

## Discussion

A major research objective for biomedical research is the detection of genetic factors involved in human disease at multiple levels including variation, gene expression and cellular roles. The evolutionary perspective of human disease is less appreciated, compared to the functional genomics of human genes and proteins, by either computational or experimental means. Combining evolutionary characters to structural features such as IDR presence which has yet to be systematically studied in conjunction with specific disease classes, can provide a novel analysis framework of the human genome with respect to disease.

In this study, we report a genome-wide analysis of the compositional bias association with disease in human proteins and their taxonomic distribution. It is the first time that a combined genome-wide analysis of these aspects is reported, from various structural, functional and evolutionary angles. Our analysis includes novel views on the relation between compositional bias and disease-association, demonstrating a strong correlation between the two features. Delving deeper into the contribution of specific amino acids to compositionally biased regions of disease-associated genes across the human genome, we demonstrate that charged, hydrophilic residues are over-represented in genes with confident disease associations.

We adopt a comparative genomics perspective for the evaluation of disease association of compositional bias in human proteins, using a curated list of 100 human proteins, as a first step towards this direction in a controlled manner. We delineate conservation patterns of the annotated gene set across taxonomic categories, taking advantage of the great plethora of sequenced genomes across the tree of life, using a total of 11297 representative proteomes.

The described framework of structurally and functionally annotated gene queries against multiple species has been developed with the view of future directions, encompassing the entire human genome and all known gene-disease associations. This will potentially allow us to elucidate specific evolutionary patterns of groups of genes involved in the same disease, serving as a tool to better understand the underlying mechanisms and identify appropriate model organisms for experimental investigation.

## Data Availability

All data underlying the analyses are available as part of the article or as referenced external data sources and no additional source data are required. Zenodo: Phylogenetic profile of 100 annotated low complexity proteins against the Uniprot Reference Proteome dataset. https://doi.org/10.5281/zenodo.7486339 ( Chasapi, 2022). This project contains the following extended data:
- cb100-query-20221223.map (The phylogenetic profile of the 100 selected annotated low complexity proteins against the Uniprot Reference Proteome dataset) Data are available under the terms of the Creative Commons Attribution 4.0 International license (CC-BY 4.0).
