# Supplementary material for: Disease association and comparative genomics of compositional bias in human proteins
Source: F1000Res. 2023 Apr 14;12:198. Originally published 2023 Feb 20. [Version 2] doi: 10.12688/f1000research.129929.2 (PMC10111144; doi:10.12688/f1000research.129929.2)
Supplement: Peer review history

# Peer review: Reviewer response for version 2

**DOI:** 10.5256/f1000research.146550.r169726

**Published:** 2023-04-17

**Authors:** Stella Tamana

**Affiliations:** 1 Molecular Genetics Thalassaemia Department, The Cyprus Institute of Neurology and Genetics, Nicosia, Cyprus

The authors have addressed my comments/recommendations and I have no further comments to make.

Is the work clearly and accurately presented and does it cite the current literature?

Yes

If applicable, is the statistical analysis and its interpretation appropriate?

Yes

Are all the source data underlying the results available to ensure full reproducibility?

Yes

Is the study design appropriate and is the work technically sound?

Yes

Are the conclusions drawn adequately supported by the results?

Yes

Are sufficient details of methods and analysis provided to allow replication by others?

Yes

Reviewer Expertise:

Bioinformatics, Compositionally Biased Regions, Comparative genomics

I confirm that I have read this submission and believe that I have an appropriate level of expertise to confirm that it is of an acceptable scientific standard.

# Peer review: Reviewer response for version 2

**DOI:** 10.5256/f1000research.146550.r169727

**Published:** 2023-04-17

**Authors:** Gregory Amoutzias

**Affiliations:** 1 Bioinformatics Laboratory, Department of Biochemistry and Biotechnology, University of Thessaly, Larissa, Greece

The authors have addressed my suggestions/corrections and I have no further comments to make.

Is the work clearly and accurately presented and does it cite the current literature?

Partly

If applicable, is the statistical analysis and its interpretation appropriate?

Yes

Are all the source data underlying the results available to ensure full reproducibility?

Yes

Is the study design appropriate and is the work technically sound?

Yes

Are the conclusions drawn adequately supported by the results?

Yes

Are sufficient details of methods and analysis provided to allow replication by others?

Yes

Reviewer Expertise:

Bioinformatics, evolution, sequence analysis

I confirm that I have read this submission and believe that I have an appropriate level of expertise to confirm that it is of an acceptable scientific standard.

# Peer review: Reviewer response for version 1

**DOI:** 10.5256/f1000research.142650.r164958

**Published:** 2023-03-13

**Authors:** Gregory Amoutzias

**Affiliations:** 1 Bioinformatics Laboratory, Department of Biochemistry and Biotechnology, University of Thessaly, Larissa, Greece

The authors identified disordered regions in human proteins, they investigated their annotation and their association with diseases and then investigated the presence/absence pattern of these disordered proteins in homologs from various taxa. Interestingly, the authors found that disease-related proteins tend to have LCRs that are enriched in charged, hydrophilic amino acids. In addition, they investigated the phylogenetic distribution of these proteins. The authors discuss in detail some very interesting disease-associated genes with LCRs and their distinct phylogenetic depth. This is an interesting and very useful study that sheds more light (also from an evolutionary point of view) on the intriguing link between LCRs and disease, and importantly, it functions as a pilot study for a larger-scale planned analysis by the authors.

 Comments/suggestions:

 In introduction, I would also add a paragraph and explain in more detail the terms 'compositional bias' and 'low complexity' and explain that they are strongly linked to IDRs. Concerning the LCRs part, I would include the references of Wooton *et al.* (1994 ^1^), of Karlin *et al.* (2002 ^2^), of Schaper *et al. *(2014 ^3^) and explain that they were originally thought of as junk or linker regions, but not anymore (Haerty *et al.*, 2010 ^4^).

 In Methods: “Searching with query datasets against Proteomes for the creation of phylogenetic profile patterns was performed with DIAMOND blastp”. I understand that the authors search for homologs and not only orthologs, is that correct? Also, concerning the cutoff of 21% sequence similarity, is it for any local alignment that DIAMOND reports, or is it for a certain query coverage too?

 Maybe Figure 1 and Table 1 could be moved to Results.

 In Results, third paragraph. The authors clearly identify a strong compositional bias for disease-associated transcripts. It would be very informative to mention the enrichment fold of 1.8 ((1845/1780) / (36250/62827)). Also, better mention the p-value as 1e-5. I wonder if disease-associated genes tend to have more transcripts on average than non-disease genes? If they do, are the transcripts-isoforms more frequently retaining the compositionally biased region? Maybe the authors could repeat this chi-square test, where they use the longest transcript per gene.

 Some thoughts for the future, when the authors perform their planned large-scale analysis: Do disease-associated genes and transcripts with compositional bias have more wide or more restricted gene expression profiles?

 Results: “The striking over-representation of serine/threonine (S/T) tracts, along with glutamate/glutamine (E/Q) and proline (P) followed by lysine (K) is indicative of the main residue types that might affect functional properties of human proteins, including their potential association with known phenotypes, such as polyglutamine tracts with neurodegenerative diseases (Bunting et al., 2022)”

 It would also be informative to add that tandem repeats of short oligopeptides that are rich in glycine, proline, serine or threonine are capable of forming flexible structures that bind ligands under certain pH and temperature conditions (Matsushima *et al.*, 2008 ^5^). Maybe also add Williamson *et al. *(1994 ^6^), concerning proline rich regions.

 I would recommend that the authors explain somewhere in detail what the CAST score means.

 Concerning the section of phylogenetic profiling, first paragraph: It would be informative to show what is the enrichment of disease in the well characterized CB proteins, compared to the background, and the statistical significance (Hypergeometric test probably). For example, the background is X disease-proteins in Y total proteins (or genes) of the genome. Enrichment: (17/100)/(X/Y).

 Same section, second paragraph: I guess its pairwise local sequence alignment with DIAMOND?

 Same section, third paragraph: “Focusing on the 100-gene subset with confident disease associations”. I guess these are the 17 proteins of the 100 gene subset with known IDs?

 Within the abstract, the authors use all three related terms, intrinsically disordered regions (IDRs), compositional bias, low complexity regions (LCRs). Maybe they could use only one (in the abstract). Also, they should explain in the Introduction their inter-changeability.

 Abstract: “The evolutionary rate of disordered proteins”. I would rephrase that as disordered protein regions.

 Abstract: Low complexity proteins or proteins with LCRs?

 In keywords, I would rephrase towards: low complexity region (LCR).

 In Introduction, please correct the CAID Predictors et al reference.

 In Introduction: “are transiently associated with intrinsic disorder in nucleated cells”. Could the authors please explain in more detail what they mean with the term “transiently”?

 Introduction: the paragraph that discusses the link between intrinsic disorder and disease: It would be nice to briefly mention one specific example of how intrinsic disorder is associated with human disease.

 Introduction: “while others appearing particularly diversified”. Maybe the term “rapidly evolving” would be better.

 In Methods, when first mentioning GHR, I would include the entire name “Genetics Home Reference”.

 In Methods: “a total of 7269 disease-gene, high-confidence associations”. It would be even more informative if the number of unique genes and the number of unique diseases in these associations were included too.

 In Methods, could the authors please elaborate more on what MagicMatch does? I did not exactly understand this part: “to verify the identity of the reference proteome collection against the modified identifier space”.

 Concerning the calculation of amino acid frequencies across the Ensembl protein set, was that done for all protein isoforms of a certain gene, or only for one representative isoform (i.e. the longest one) from each gene?

 In table 1, the numbers are for human genes or transcripts/protein isoforms?

 In Methods: “For each visualisation, a list of the NCBI IDs”. Which types of IDs?

 Results: “(GRCh38.p13), containing 119068 gene transcripts”. Could the authors also mention the number of genes?

 Results: “Examination of the low complexity gene dataset features highlighted the significant divergence among amino acid related, low complexity frequencies.” Maybe better use the term difference because divergence relates to conservation.

 Results: “Charged, hydrophilic residues appear over-represented”. I would also mention them in parenthesis.

 In figure 3, this must be the subset of 17 disease-associated genes from the set of 100 human proteins with well-characterised compositionally biased regions? . The title of Figure 3 should be changed accordingly. Is it possible to also include a key of DOIDS-disease next to the figure, or is it too many of them?

 Figure 4 legend: could the authors explain this more?: “The heatmap range reflects the dissimilarity matrix of the plotted values.”. I am not sure I understood figure 4. Does the figure only show if a homologue is present in a certain species, or does it show as well if the homologue also contains an LCR or CB region as well? Does this correspond to the red/blue colour of the matrix?

 Concerning figure 4, as a thought/suggestion for future studies, when the authors move to larger-scale analyses, maybe they could also include an analogous analysis, where they show the presence of orthologs, not homologs. For that, they would have to use best reciprocal blast and one representative protein (the longest) from each gene of a genome. Or, maybe the authors could do that using the orthology presence from the OMA database (https://omabrowser.org/oma/home/).

 Figure 5: visualized by Lifemap.

 Concerning supplementary .map file, I would simply convert it to a csv file for import in excel. I would also add two columns, one with the species name of the proteome and another one with the wider taxonomic group that the species belongs to. Also, could the authors explain what the numbers in the cells correspond to, I guess it's the number of homologs in that species?

Is the work clearly and accurately presented and does it cite the current literature?

Partly

If applicable, is the statistical analysis and its interpretation appropriate?

Yes

Are all the source data underlying the results available to ensure full reproducibility?

Yes

Is the study design appropriate and is the work technically sound?

Yes

Are the conclusions drawn adequately supported by the results?

Yes

Are sufficient details of methods and analysis provided to allow replication by others?

Yes

Reviewer Expertise:

Bioinformatics, evolution, sequence analysis

I confirm that I have read this submission and believe that I have an appropriate level of expertise to confirm that it is of an acceptable scientific standard, however I have significant reservations, as outlined above.

## References

[1] : Non-globular domains in protein sequences: automated segmentation using complexity measures. *Comput Chem* .1994;18(3) : 10.1016/0097-8485(94)85023-2 269-85 10.1016/0097-8485(94)85023-2 7952898

[2] : Amino acid runs in eukaryotic proteomes and disease associations. *Proc Natl Acad Sci U S A* .2002;99(1) : 10.1073/pnas.012608599 333-8 10.1073/pnas.012608599 11782551PMC117561

[3] : Deep conservation of human protein tandem repeats within the eukaryotes. *Mol Biol Evol* .2014;31(5) : 10.1093/molbev/msu062 1132-48 10.1093/molbev/msu062 24497029PMC3995336

[4] : Low-complexity sequences and single amino acid repeats: not just "junk" peptide sequences. *Genome* .2010;53(10) : 10.1139/g10-063 753-62 10.1139/g10-063 20962881

[5] : Flexible structures and ligand interactions of tandem repeats consisting of proline, glycine, asparagine, serine, and/or threonine rich oligopeptides in proteins. *Curr Protein Pept Sci* .2008;9(6) : 10.2174/138920308786733886 591-610 10.2174/138920308786733886 19075749

[6] : The structure and function of proline-rich regions in proteins. *Biochem J* .1994;297 ( Pt 2)(Pt 2) : 10.1042/bj2970249 249-60 10.1042/bj2970249 8297327PMC1137821

# Peer review: Reviewer response for version 1

**DOI:** 10.5256/f1000research.142650.r164957

**Published:** 2023-03-03

**Authors:** Stella Tamana

**Affiliations:** 1 Molecular Genetics Thalassaemia Department, The Cyprus Institute of Neurology and Genetics, Nicosia, Cyprus

The presented manuscript “Disease association and comparative genomics of compositional bias in human proteins” focuses on the very interesting, but challenging to analyze and interpret, study of the structural, functional and evolutionary properties of compositional bias in human proteins. The authors report novel findings and a strong correlation between compositional bias and disease association. Studying compositional bias and disease association, both computationally and experimentally, has been quite challenging over the years, mainly because proteins with compositional bias tend to conform into non-globular structures or be in a disordered state and thus, requiring special treatment in fundamental steps of comparative genomic analyses and experimental procedures to determine their three-dimensional (3D) structures. This is the first time that, successfully, a computational framework, combined genome-wide disease-association analysis of compositional bias with regards to their functional, structural, and evolutionary properties. The presentation is very clear, well-structured, and easy to comprehend. The reported computational framework and methodology take advantage of up-to-date computational tools and statistical packages. This paper is of interest to scientists within the field of human diseases and variant interpretation as elucidating specific functional and structural patterns of groups of genes (especially those presenting extreme compositional bias) involved in the same disease will ultimately help discover the underlying mechanisms and develop new experimental procedures. 

**Minor comments:**

- **Introduction – Disordered proteins exhibit specific patterns at the sequence level**
  - This section will be greatly enhanced if the authors consider adding a paragraph (or 2-3 lines) of the structural characteristics and underlying mechanisms of IDPs/IDRs and thus, giving the reader the opportunity right from the start of the paper to understand the correlation between compositional bias and disease-association. For example, providing in a bit more detail that order-promoting residue types are commonly found within the hydrophobic cores of foldable proteins as opposed to disorder-promoting residues typically located at the surface of foldable proteins (for references see Theillet *et al.*, 2013 ^1^; Uversky, 2013 ^2^). Also, another example could be that hydrophobic enriched regions are prone to induce either self-aggregation or/and intermolecular interactions with surrounding proteins when exposed and thus, trigger aggregation (for reference see Grignaschi *et al.*, 2018 ^3^). 

Line 1-3: please consider adding references.

Is the work clearly and accurately presented and does it cite the current literature?

Yes

If applicable, is the statistical analysis and its interpretation appropriate?

Yes

Are all the source data underlying the results available to ensure full reproducibility?

Yes

Is the study design appropriate and is the work technically sound?

Yes

Are the conclusions drawn adequately supported by the results?

Yes

Are sufficient details of methods and analysis provided to allow replication by others?

Yes

Reviewer Expertise:

Bioinformatics, Compositionally Biased Regions, Comparative genomics

I confirm that I have read this submission and believe that I have an appropriate level of expertise to confirm that it is of an acceptable scientific standard.

## References

[1] : The alphabet of intrinsic disorder: I. Act like a Pro: On the abundance and roles of proline residues in intrinsically disordered proteins. *Intrinsically Disord Proteins* .2013;1(1) : 10.4161/idp.24360 e24360 10.4161/idp.24360 28516008PMC5424786

[2] : The alphabet of intrinsic disorder: II. Various roles of glutamic acid in ordered and intrinsically disordered proteins. *Intrinsically Disord Proteins* .2013;1(1) : 10.4161/idp.24684 e24684 10.4161/idp.24684 28516010PMC5424795

[3] : A hydrophobic low-complexity region regulates aggregation of the yeast pyruvate kinase Cdc19 into amyloid-like aggregates in vitro. *J Biol Chem* .2018;293(29) : 10.1074/jbc.RA117.001628 11424-11432 10.1074/jbc.RA117.001628 29853641PMC6065187
